# Lifestyle interventions in the maritime settings: a systematic review

**DOI:** 10.1186/s12199-020-00848-7

**Published:** 2020-03-31

**Authors:** Fereshteh Baygi, Shirin Djalalinia, Mostafa Qorbani, Masoumeh Dejman, Jesper Bo Nielsen

**Affiliations:** 1 Centre of Maritime Health and Society, Department of Public Health, University of Southern Denmark, Esbjerg, Denmark; 2 Non-communicable Diseases Research Center, Endocrinology and Metabolism Population Sciences Institute, Tehran University of Medical Sciences, Tehran, Iran; 3 Deputy of Research and Technology, Ministry of Health and Medical Education, Tehran, Iran; 4 Non-communicable Diseases Research Center, Alborz University of Medical Sciences, Karaj, Iran; 5 Amarex Clinical Research, LLC, Germantown, USA; 6 Research Unit of General Practice, Department of Public Health, University of Southern Denmark, Odense, Denmark

**Keywords:** Maritime settings, Interventions, Systematic Review

## Abstract

**Background:**

Evidence on workplace health promotion interventions at sea is scattered and includes different methodological approaches. The continued focus on lifestyle and health promotion on land-based industries makes it pertinent to evaluate available data from maritime settings to gain systematic knowledge on the field.

**Methods:**

In this systematic review, we systematically searched PubMed and NLM Gateway (for MEDLINE), Institute of Scientific Information/Web of Science (ISI/WOS), and SCOPUS up to January 2019 using standard keywords including lifestyle interventions in the maritime setting. Two independent reviewers assessed papers and extracted the data. The quality of included studies was assessed using the Cochrane Risk of Bias tool. Due to significant heterogeneity between studies, the effectiveness of interventions was presented as a qualitative synthesis.

**Results:**

After the initial search and refinement based on a total of 4432 records, ten articles met eligibility criteria and were included in the final review. Six studies originated from US maritime settings, 3 studies were conducted on Danish seafarers and one study came from Finland. The main focus of 6 studies was educational interventions including stress management, healthy eating, anti-smoking and anti-drinking sessions, sexual behavior program, and advice about preventive strategies. Four studies described the implementation of interventions, including micro-nutrient supplementation, physical activity, and pharmacotherapy. Follow-up assessments occurred over a time period ranging from 80 days to 2 years. Three studies found a positive though limited effect of structural and/or education interventions in maritime settings. The quality of all included studies was modest.

**Conclusion:**

Results of this systematic review show that a limited number of studies of lifestyle interventions in the maritime setting exist and that the quality of them is generally modest. Also, most of the interventions identified have failed to demonstrate substantial health benefits for seafarers.

Systematic review registration number in PROSPERO: CRD42019134533

## Background

Cardiovascular disease (CVD) is the leading cause of death in seafarers [1]. A study conducted among Danish seafarers revealed that cardiovascular risk factors such as obesity, high blood pressure, and increased levels of triglycerides are highly prevalent [2]. Likewise, a study among Iranian seafarers showed that the prevalence of metabolic syndrome and excess weight was 15% and 51%, respectively [3]. Moreover, the prevalence of behavioral risk factors like smoking and physical inactivity in seafarers is high [2–4]. Ship-specific stress situations, lack of sufficient and appropriate exercise, and malnutrition have been described as the main risk factors for CVD in the maritime setting [1]. An unhealthy lifestyle may have different consequences such as ill-health, absence due to sickness and loss of productivity [5]. Worksites—even in land-based occupations—represent a major venue for influencing the overall health of workers [6]. Since seafarers often for several months have a second home on board ships, unhealthy lifestyle together with specific working conditions may in such a microcosmos form a hazardous environment not only affecting the health and wellbeing of the seafarer but also potentially affecting the economy and safety of the ship. So, it is important to target the health behavior of seafarers on board as a method for future prevention programs.

The present paper summarizes and evaluates existing interventions targeting the health and wellbeing of seafarers. It is expected that this study will provide relevant input for health policymakers in this maritime population to develop health promotion strategies and can inspire other researchers to fill the gap of the presently limited quality research in the field of health promotion at sea.

## Methods

### Identification of relevant studies

**Fig. 1:**
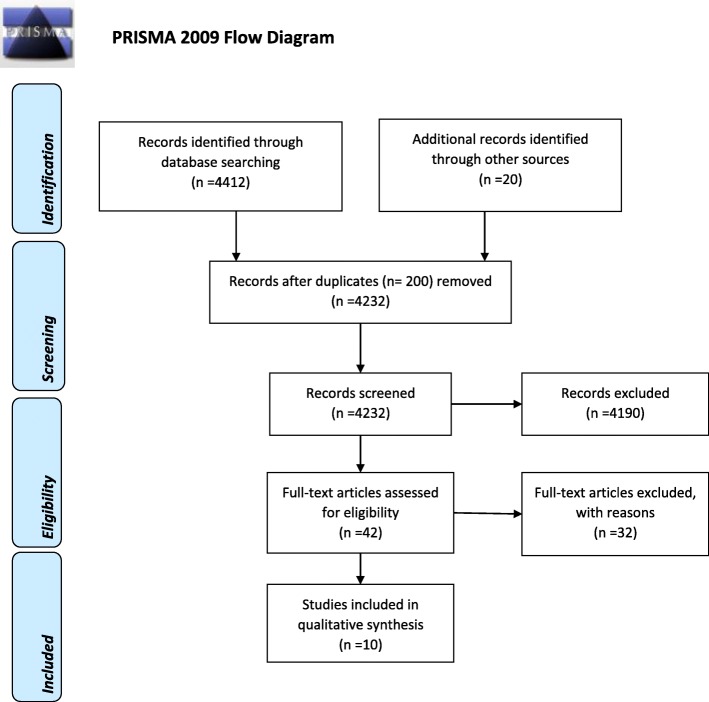
PRISMA 2009 flow diagram

This systematic review was carried out to identify and assess all evidence on the lifestyle interventions in seafarers. The PRISMA-P guideline was used to develop the current systematic review (Fig. 1) [7], the items for meta-analysis were not applicable for this review. All the documents are based on the details of the study protocol. The registration number of our study in the International Prospective Register of Systematic Reviews (PROSPERO) is CRD42019134533.

The search terms developed concentrating on two main roots of “seafarers” and “health interventions”. There was no limitation for language and time of papers. All studies carried out until the end of January 2019 were considered in this systematic review. For documents other than English, the necessary arrangements were taken for their translation. To assess the optimal sensitivity of searching for documents, we simultaneously searched the most comprehensive related databases of PubMed and NLM Gateway (for MEDLINE), Institute of Scientific Information, Web of Science (ISI/WOS), and SCOPUS as the main international electronic data sources (Appendix 1). Moreover, some reference lists of included studies or relevant reviews through the search were scanned to identify potentially eligible studies.

### Inclusion and exclusion criteria

To obtain a comprehensive overview of the works that have been done so far, we focused on all studies published up to January 2019—on the lifestyle changes including type and quality of food, vitamin/mineral supplementation, physical activity, and educational programs, etc. Studies with outcomes such as smoking, stress level, physical activity, alcohol consumption, body mass index, behavior change, and other outcomes complying with the objective of the study in the target group were included. Also, we included all observational studies. All relevant results were extracted from randomized control trials, quasi-experimental studies, and non-randomizes studies. There was no limitation for the target groups in terms of age and gender and language of published studies. Duplicate citations and non-peer-reviewed publications were excluded. Book chapters and available conference proceedings were also considered.

### Quality assessment and data extraction

All systematic processes of literature searches, quality assessment, and data extraction of eligible papers were conducted by two independent research experts and any discrepancy between them was resolved through referencing a third expert opinion.

We investigated the quality of the included randomized controlled trials (RCTs) using the Cochrane Back Review Risk of Bias criteria [8]. The criteria list consists of 11-items evaluating internal validity. We also used The Joanna Briggs Institute Critical Appraisal tool for Quasi-Experimental Studies (non-randomized experimental studies) to assess the quality of quasi-experimental (before–after) studies [9]. This checklist has 9 items and the total score was the number of positive items (range 0–9 scores).

Two independent investigators scored items as positive when they met the criteria, negative if they did not, or as inconclusive if there was insufficient information.

The extracted data included: author and year of publication, population characteristics (mean age/age range and subjects), methodological characteristics (study design, period of the study, sample size, type of ship or shipping sector, type of interventions, outcomes and outcome measurement).

Data synthesis was the main strategy. The number of included studies was more than 4 studies but the heterogeneity of them in terms of the type of interventions, follow-up period, randomization, etc. hampered the possibility of a meta-analysis.

### Statistical analysis

Due to heterogeneity between studies in terms of study design, type of intervention, study duration, and different outcome measurements, the results were presented as a qualitative synthesis.

## Results

### Study selection process

A total of 4432 studies were identified following the initial search. After the removal of 200 duplicates, 4232 records remained. A total of 4190 articles did not meet selection criteria, so they were excluded after screening titles and abstracts. Of the remaining 42 studies retrieved, 32 were excluded after the full-text review because the outcomes did not comply with the objectives of the current study (Fig. 1). Finally, 10 articles met the inclusion criteria in the review [10–19].

### Study characteristics

A summary of all included studies is shown in Table 1. Four studies were RCTs, three were non-randomized studies and three were quasi-experimental. The studies were done in three countries. Six studies were done in the US maritime setting, three were conducted among Danish seafarers, and one in Finland.

**Table 1:** Characteristic of the included studies

Author, Year, of publication, Country	Study design	Study subject	Study period	Sample size	Mean age/ Age range	Setting (type of ship, shipping sector)	Type of intervention	Outcome measurement	Outcome	Groups	Results & Significancy	Quality score
Jepsen [16], 2016, DK	NRS	Seafarers	2 years	141	41.3	NP	-Specific advice regarding treatment for Metabolic Syndrome -Specific advice regarding preventive measures	Medical examination	-BMI -WC -MetS -Smoking -Alcohol consumption	-----	Increment in all outcomes (NS)	3 of 9
Hjaroe [14], 2014, DK	NRS	Seafarers	1 year	Q: 49 F: 193	NP	Cargo service company	-Cooking course	-Qualitative study -Self-report questionnaire	-Opinion about the course -Subsequent changes in promoting a healthy diet at sea -Eating healthy	-----	-Increment in knowledge from the intervention course -Increment in serving fruits and vegetables -Increment in applying fat and sugar reducing tips Increment in eating healthy (S)	3 of 9
Gasier [17], 2014, USA	RCT	Submariners	1 year	53	**•Placebo:** 28.3 **Experiment**_**1**_**:** 29.4 **Experiment**_**2**_**:** 28.1	Submarine	Vitamin D supplementation	-Medical examination	-25(OH)D -Osteocalcin	•Placebo •Trial_1_ •Trial_2_	-Increment of 25 (OH)D in all groups (S) -Increment of osteocalcin in all groups (S)	7 of 11
Hjarnoe [10], 2013, DK	QE	Seafarers	1 year	606	41	Cargo service company	-Healthy cooking courses for ships cooks -Improvement of fitness facilities -Smoking cession courses -Individual guidance -Extra health check ups	-Self-administrated questionnaire -Individual health profile	-Smoking -Physical activity -Sugar intake -Metabolic syndrome	Single-group pre-post design	-Reduction in smoking (NS) -Increment in Physical activity ≥3 times weekly at home and at sea (NS) -Increment in Physical activity <1 weekly or never at home (NS) -Reduction in Physical activity <1 weekly or never at sea (NS) -Reduction in overeating at home and at sea (NS) -Reduction in sugar intake at home and at sea (S) -Reduction in Metabolic syndrome (S) -Increase in physical fitness scores (S)	5 of 9
William [13], 2010, USA	QE	Navy members	NP	142	**Officer** 41.1 **Sailor** 29.5	Navy	Web-enhanced behavioral self-management program for stress	Questionnaire	Perceived stress levels	-----	Reduction in perceived stress levels (S)	4 of 9
Duplessis [18], 2005, USA	RCT	Sailor	76 days	51	28	Submarine	Vitamin D supplementation	Medical examination	-Calcium -Phosphate -25(OH)_2_ D -1,25(OH)_2_ D -Alkaline Phosphatase -Osteocalcin	•Control •Trial	-No change in calcium -Increment in phosphate (NS) -Reduction in 25(OH)_2_ D (S) - Increment in 1,25(OH)_2_ D (NS) -Reduction in alkaline Phosphatase (S) -Increment in osteocalcin (S)	4 of 11
Swanson [12], 2003, USA	RCT	Sailor	12 months	131	27.1	Navy ship	-Pharmacotherapy -Tobacco cessation group program	-Qualitative study (Self-report)	Continuous abstinence of smoking - Not smoking at 6 and 12 months - Not smoking at 6 months, but smoking at 12 months - Smoking at 6 months but not smoking at 12 months -Still smoking at 6 and 12 months	•Nicotine patch •Bupropion •Bupropion and nicotine patch •Control	-No significant differences between groups	4 of 11
Saarni [11], 2001, Finland	QE	Sailors	1 year	392	42	-Cargo ship -Passenger cruise ferry	**Educational program about:** -Food preparation on board -Serving of meals on board -Over-weight reduction -Increasing physical fitness -Anti-smoking, anti-drinking -Rehabilitation	Re-questionnaire and qualitative study	**Behavior change in:** -Physical activity -Smoking -Alcohol consumption -Preparing healthier food -Mental stress -Work satisfaction	-----	-Increment in physical activity (NP) -Reduction in smoking (NP) -Reduction in alcohol consumption (NP) -Increment in preparing healthier food (NP) -Reduction in mental stress (NP) -Increment in work satisfaction (NP)	3 of
Booth-Kewley [15], 2001, USA	RCT	Marines	1 year	176	**•Intervention**: 23.11 **•Control:** 23.28	Marine Crops	STD/HIV-intervention program	Qualitative study (Telephone interview)	-Sexual behavior **-**Alcohol consumption	•Intervention •Control	-Increment in using condom in intervention group compared to Control (S) *-* Increment in discussion with partner about condom in intervention group compared to Control (NS) - Increment in discussion with partner about their sexual history in intervention group compared to Control (S) -Reduction in alcohol consumption in intervention group compared to Control (NS)	3 of 11
Bennett [19], 1985, USA	NRS	Crew members	8 weeks	19	**•Trial:** 29.33 **•Control:** 25.67	Nuclear Submarine	Exercise test	-Especial medical and anthropometry measurements	-Weight -BF% -VO2 max (O2 consumption)	•Trial •Control	-Reduction in weight and BF% in both groups (NS) -No change in VO2 max in trial group -Reduction in VO2 max in control group	5 of 9

NRS: Non-Randomizes Study, RCT: Randomized clinical trial, QE: Quasi-Experimental, Q: Qualitative, NP: Not provided, F: Follow up, NS: non-significant, S: Significant, BMI: Body mass index, BF: Body fat, WC: Waist circumference, MetS: Metabolic Syndrome, STD: Sexually transmitted diseases

Main focus of 6 studies was educational interventions including stress management [13], healthy eating [14], anti-smoking and anti-drinking sessions [10, 11], sexual behavior program [15], and advice about preventive strategies [16]. Of these 6 studies, four were conducted on multi-component interventions [10, 11, 15, 16]. Practical interventions including micro-nutrient supplementation [17, 18], exercise test [19], and pharmacotherapy [12] were implemented in the four studies.

Only one study done on seafarers with metabolic syndrome (MetS) was basically a treatment intervention [16]. The content of all the other 9 studies was based on prevention. Follow-up assessments occurred over a time period ranging from 80 days to 2 years with different time intervals. Three studies included more than one intervention. There were 2 studies including vitamin D supplementation to submariners [17, 18]. One study was a web-based educational program [13].

### Qualitative synthesis

- Dietary habits and factors

Five studies assessed multi- or single-component interventions to improve diet: preparing healthier food [11], avoiding overeating and high-sugar product intake [10], eating behavior on board [14], and maintenance of vitamin D status [17, 18].

After training for ship cooks in preparing healthier meals, the meals at the 1-year follow-up got healthier compared with baseline [11]. A significant decline was observed in the intake of high-sugar products, but no significant change was found in overeating habits 1 year after delivering healthy cooking courses for ship cooks [10]. In another 1-year follow-up study, a training intervention for ship cooks made a significant change in the self-reported eating behavior on board [14]. Two studies examined the effect of vitamin D supplementation on the maintenance of 25(OH)D serum levels in submariners [17, 18]. A 400-IU/day vitamin D supplementation was insufficient in maintaining serum vitamin D levels in underway submariners [18]. A significant benefit for the submariners supplemented with 1000 or 2000 IU/day vitamin D over the non-supplemented ones was not observed following a 3-month patrol, even though the submariners who were supplemented with 2000 IU/day of vitamin D3 did experience the largest positive change in mean serum 25(OH)D levels [17].

- Physical activity

Three studies addressed physical activity outcomes measured as physical exercise level [10, 11], physical fitness score [10], and maximal oxygen consumption (VO2max) [19].

Mild [10] to moderate [11] improvement in exercise level and a significant increase in physical fitness scores [10] were noted after implementing both structural and education interventions at the maritime workplace. Following physical training and deconditioning, VO2max remained constant in the intervention group but had declined, though statistically insignificant, by 7% in the control group [19].

- Cardio-metabolic risk factor

Five interventional studies addressed changes in cardio-metabolic risk factors including MetS, body mass index (BMI), waist circumference (WC) [10, 16], and weight and body fat (BF) as an outcome [19]. After the delivery of a multicomponent intervention, a significant decrease in the percentage of seafarers with MetS was measured [10]. In contrast, another study including general and specific advice regarding treatment or preventive strategies for MetS showed an increase in all outcomes such as BMI, WC, and the prevalence of MetS in the studied group at follow-up [16].

- Smoking and alcohol habits

Five studies examined the effect of interventions on smoking [10–12, 16] or alcohol drinking habits [11, 15, 16].

In one of three studies that implemented only advice-based anti-smoking interventions, a reduction in the number of smokers was reported following a 1-year period [10, 11, 16]. In a RCT, where three various pharmacotherapies for smoking cessations were compared with a control group, no significant differences in smoking habits were detected between groups. In all four groups, nearly 70% of the smokers were still smoking at 6 and 12 months [12].

Among three studies that assessed change in alcohol drinking habits following educational intervention, two studies found no significant change [15, 16], whereas the third reported a reduction, but did neither provide any quantitative information about the decline nor any statistics [11].

- Stress and work satisfaction

Two studies examined interventions aimed at reducing stress levels and both reported a positive outcome of the intervention [11, 13]. In the study that implemented a web-enhanced behavioral self-management program against stress, this reduction was statistically significant [13]. In another interventional study aimed at work satisfaction, an increment in this outcome after providing health education for the seafarers was observed [11].

- Risky sexual behaviors

An intervention study showed few positive changes in the sexual behavior of the mariners after delivering a STD/HIV intervention program course [15].

### Quality assessment

The results of the quality assessment are presented in Table 1. One out of four RCTs (25%) fulfilled six or more of the quality items; the scores ranged from 3/11 to 7/11, a moderate risk of bias. Lack of intervention allocation, concealment, and intention-to-treat analysis were identified in all RCTs. For six non-randomized studies, the quality scores ranged from 3/9 to 5/9; none of them met six or more of the quality items.

## Discussion

Our review included ten intervention studies focused on changing lifestyle behaviors and health outcomes through structural and/or educational interventions in seafarers. Assessed outcomes were classified into six categories including dietary habits and factors, physical activity, cardio-metabolic risk factors, smoking and alcohol habits, stress and work satisfaction, and risky sexual behaviors. Some of the included studies reported a significant positive effect of implemented interventions, although the positive changes were limited.

Overall, the results of this systematic review showed that most of the intervention studies conducted on lifestyle behaviors and health outcomes at the maritime workplace were methodologically weak and were not well designed and conducted. However, as an overall assessment, the evidence tends to suggest that lifestyle interventions are feasible and may have the potential to improve health behaviors in this high-risk group of seafarers. For comparison, the first review on the behavioral lifestyle interventions for nurses also reported that all included studies had limitations and high risk of bias, but later found benefits for the outcomes including smoking habits, fat mass and physical fitness [20].

In this review, three studies found beneficial effects following educational and/or structural interventions focusing on the seafarers’ dietary behavior [10, 11, 14]. Two studies found multi-component physical activity interventions to be effective in increasing seafarers’ physical activity and/or fitness [10, 11]. Most interventions were non-effective regarding weight-related indicators. A review of 15 systematic reviews on land-based workplace interventions targeting diet and/or physical activity reported that almost all interventions made small but significant changes in physical activity, fitness, dietary behavior or weight, and found that intervention involving multi-component programs tended to be more successful [21].

A healthy diet during working time is less available at sea due to restricted space for storage and lack of proper equipment, high prices for fresh fruit and vegetables, and low frequency of supply options on board of the ships [14]. In other occupational settings, fruit and vegetable interventions are generally successful. For example, a worksite study conducted in Denmark resulted in an average increase of 1 serving of fruit and vegetables per day for each person [22]. A review study with the aim of identifying efforts to improve fruit and vegetable intake at workplace revealed that organizational support, workers participation, targeting multiple health behaviors are some factors which play a significant role in the success of such a program [23] which researchers should consider in order to enhance the effectiveness of interventions in maritime settings.

Seafarers are often sedentary on board; space and physical capacities on board are confined for the exercise during leisure time. Besides, technological advancements in modern vessels have removed much physical labor and caused more physical inactivity among seafarers [10].

Seafarers are frequently characterized in the literature as heavy smokers and drinkers [24]. In our systematic review, two out of 4 studies reported a small reduction in tobacco consumption [11, 12]. Results from a Cochrane review of interventions for smoking cessation at various workplaces provided strong evidence that group therapy programs, individual and group counseling, multiple intervention programs aimed mainly or solely at smoking cessation, and pharmacotherapies significantly increased the likelihood of quitting smoking [25]. However, in the case of the onboard setting, the physical environment, operational demands, and psychosocial issues may provide obstacles for a successful tobacco cessation program [12]. Therefore, a more specifically tailored approach is probably required, which takes into account specific conditions in the maritime settings.

Submariners experience a reduction in their serum vitamin D [25(OH)D] levels due to sunlight deprivation and may take advantage of vitamin D supplementation to maintain vitamin D status [17]. However, the results of two placebo-controlled studies failed to provide any robust support to the efficacy of daily vitamin D supplementation on the maintenance of serum vitamin D [25(OH)D] levels during a submarine patrol [17, 18]. Some potential confounders, such as subject compliance and insufficient prescribed dose of vitamin D may, however, have affected the findings.

One out of two intervention studies aimed at preventing or treating MetS detected a significant decrease in the percentage of seafarers with MetS [10]. Multi-dimension lifestyle interventions that target all risk factors for MetS should be implemented appropriately at the maritime settings to reduce the incidence as well as prevalence of MetS [10, 16].

A significant reduction is observed for stress levels after delivering a web-enhanced behavioral self-management program in a military setting [13]. In two other studies, the computer-based intervention was compared with in-person intervention for reducing stress in workers. One of them reported that stress levels were significantly lower in the in-person group after intervention in comparison to the computer-based intervention group [26]. In the other study, no significant difference between the two groups was observed [27]. The researchers suggest that further studies need to be done to compare the effect of equivalent computer-based program and in-person ones [28]. Despite the opposing findings, the computer-based interventions through specific features can be delivered to a large number of individuals at distance and in a cost-efficient manner [13] and might be a good approach for implementing interventions in maritime settings.

### Limitations, challenges, and strengths

The included studies had multiple limitations including small sample size, short-term follow-up, lack of proper control groups or use of inappropriate control groups, inappropriate/insufficient interventions, high drop-out rates, partial implementation failure, and insufficient outcome measures.

Researchers face specific practical challenges related to the maritime workplace setting; it is hard to have a control group technically due to the “moving nature” of work at the maritime setting [10]. Crew members shift between ships regularly, thus making a fixed assignment of crews/ships to an intervention or control condition not feasible. Assignment of a whole area in the shipping industry instead of crews/ships to a study group should be considered.

High numbers of “lost to follow-up” is another serious challenge encountered by intervention studies that can result in selection bias; probably as a result of the “moving nature” of the maritime workplace and a higher between-ship mobility among seafarers [10, 12].

To the best of our knowledge, the current study was the first systematic review conducted on this topic, which can provide sufficient evidence for further health promotion studies in this setting.

### Suggestions

Making successful lifestyle changes to promote health in a high-risk group such as seafarers seems to require multi-component interventions that well address the working and living conditions on board like psychosocial issues. Moreover, using health promotion theory or models which are fitted to this maritime setting would be useful to enhance the effectiveness and sustainability of the interventions.

And very importantly, it is a necessary factor in implementing the interventions on board ships that the shipping companies actively participate in and support the interventions. The costs paid by companies for the health interventions need to be related to the expected improved health and working capacity of the seafarers and a reduced risk of human errors on board ship.

## Conclusion

Most available studies of the feasibility and effectiveness of educational and/or structural interventions in the maritime workplace have failed to demonstrate substantial health benefits for seafarers. At present, it is not clear whether the apparently limited success of the interventions is a valid observation caused by insufficient statistical power or because the methodological quality of most interventions is generally poor. Both explanations are probably, at least in part, due to the fact that implementing interventions in the maritime workplace can be difficult and it is a challenging area for research. Studies with more rigorous designs are still needed which takes into account the specific restrictions inherent in the maritime workplace and find effective lifestyle interventions and quantify their effect on health and wellbeing and their sustainability on board.

## Data Availability

Since no dataset was generated during the current study, data sharing is not applicable.

## Appendix 1: Search strategy

**Table:**

**PubMed**	
(((Sailor[Title/Abstract]) OR seaman[Title/Abstract]) OR seafarer[Title/Abstract]) OR mariner[Title/Abstract]) OR sailer[Title/Abstract]) OR shipper[Title/Abstract]) OR seagoing[Title/Abstract]) OR navigator[Title/Abstract])))	
**Scopus**	
(( ( TITLE-ABS-KEY ( Se ) OR TITLE-ABS-KEY ( "Se" ) ) AND ( ( TITLE-ABS-KEY ( "Metabolic Syndrome" ) OR TITLE-ABS-KEY ( cardiometabolic ) OR TITLE-ABS-KEY ( " Cardiovascular Syndromes" OR TITLE-ABS-KEY ( "Diabetes Mellitus" ) OR TITLE-ABS-KEY ( "Type 2 Diabetes" ) OR TITLE-ABS-KEY ( cardiovascular ) OR TITLE-ABS-KEY ( "Syndrome X" ) OR TITLE-ABS-KEY ( "Insulin Resistance " ) OR ( TITLE-ABS-KEY ( "glucose homeostasis" ) OR TITLE-ABS-KEY ( "Homeostasis of Glucose" ) OR TITLE-ABS-KEY ( "Lipid profile " ) OR TITLE-ABS-KEY ( "lipid panel" ) OR TITLE-ABS-KEY ( "Lipid_profile" ) OR TITLE-ABS-KEY ( "Oxidative Stress" ) OR TITLE-ABS-KEY ( " inflamantion " ) OR TITLE-ABS-KEY ( " inflamantory factors " ) OR TITLE-ABS-KEY ( " antioxidant ) )) ( ( TITLE-ABS-KEY ( sailor ) OR TITLE-ABS-KEY ( seaman ) OR TITLE-ABS-KEY ( seafarer ) OR TITLE-ABS-KEY ( mariner ) OR TITLE-ABS-KEY ( sailer ) OR TITLE-ABS-KEY ( shipper ) OR TITLE-ABS-KEY ( seagoing ) OR TITLE-ABS-KEY ( navigator ) ) ) AND ( ( TITLE-ABS-KEY ( nutrition ) OR TITLE-ABS-KEY ( diet* ) OR TITLE-ABS-KEY ( salt ) OR TITLE-ABS-KEY ( sugar ) OR TITLE-ABS-KEY ( fiber ) OR TITLE-ABS-KEY ( fruit ) OR TITLE-ABS-KEY ( seagoing ) OR TITLE-ABS-KEY ( beverage ) OR TITLE-ABS-KEY ( sweet ) OR TITLE-ABS-KEY ( alcohol ) OR TITLE-ABS-KEY ( smok* ) OR TITLE-ABS-KEY ( tubaco ) OR TITLE-ABS-KEY ( "physical activity" ) OR TITLE-ABS-KEY ( exercise ) OR TITLE-ABS-KEY ( sedantry ) OR TITLE-ABS-KEY ( stress ) OR TITLE-ABS-KEY ( mental ) ) )	
**ISI/WOS**	
TOPIC: (Sailor) OR TOPIC: (seaman) OR TOPIC: (seafarer) OR TOPIC: (mariner) OR TOPIC: (sailer) OR TOPIC: (shipper) OR TOPIC: (seagoing) OR TOPIC: (navigator) ( TOPIC: (nutrition) OR TOPIC: (diet) OR TOPIC: (salt )OR TOPIC: (sugar) OR TOPIC: (fiber) OR TOPIC: (fruit)OR TOPIC: (beverage )OR TOPIC: (sweet )OR TOPIC: (alcohol)OR TOPIC: (smok) OR TOPIC: (tubaco) OR TOPIC: ("physical activity" ) OR TOPIC: (exercise) OR TOPIC: (sedantry) OR TOPIC: (stress ) OR TOPIC: (mental) Timespan=All years AND Indexes=SCI-EXPANDED, SSCI, CPCI-S, CPCI-SSH Timespan=All years	

## Abbreviations

CVD: Cardiovascular disease
PRISMA: Preferred Reporting Items for Systematic Reviews
PROSPERO: International Prospective Register of Systematic Reviews
WOS: Web of Science
RCTs: Randomized Controlled Trials
MetS: Metabolic Syndrome
BMI: Body Mass Index
WC: Waist Circumference
BF: Body Fat
STD: Sexually Transmitted Diseases
HIV: Human Immunodeficiency Virus
NRS: Non-Randomizes Study
QE: Quasi-Experimental
NP: Not Provided
NS: Non-Significant
S: Significant
